# Pollinators visit related plant species across 29 plant–pollinator networks

**DOI:** 10.1002/ece3.1051

**Published:** 2014-05-10

**Authors:** Jana C Vamosi, Clea M Moray, Navdeep K Garcha, Scott A Chamberlain, Arne Ø Mooers

**Affiliations:** 1 Department of Biological Sciences, University of Calgary2500 University Drive NW, Calgary, T2N 1N4, Alberta, Canada; 2 Department of Biological Sciences, Simon Fraser University8888 University Drive, Burnaby, V5A 1S6, British Columbia, Canada

**Keywords:** Competition, linkage rules, phylogenetic community ecology, phylogenetic signal, plant–pollinator networks

## Abstract

Understanding the evolution of specialization in host plant use by pollinators is often complicated by variability in the ecological context of specialization. Flowering communities offer their pollinators varying numbers and proportions of floral resources, and the uniformity observed in these floral resources is, to some degree, due to shared ancestry. Here, we find that pollinators visit related plant species more so than expected by chance throughout 29 plant–pollinator networks of varying sizes, with “clade specialization” increasing with community size. As predicted, less versatile pollinators showed more clade specialization overall. We then asked whether this clade specialization varied with the ratio of pollinator species to plant species such that pollinators were changing their behavior when there was increased competition (and presumably a forced narrowing of the realized niche) by examining pollinators that were present in at least three of the networks. Surprisingly, we found little evidence that variation in clade specialization is caused by pollinator species changing their behavior in different community contexts, suggesting that clade specialization is observed when pollinators are either restricted in their floral choices due to morphological constraints or innate preferences. The resulting pollinator sharing between closely related plant species could result in selection for greater pollinator specialization.

## Introduction

Pollinator specialization in communities is often discussed in terms of classic examples of evolutionary adaptations through plant–pollinator coevolution (Faegri and van der Pijl 1979; Fenster et al. 2004). Nevertheless, the observed level of pollinator specialization also has an ecological component in that it is influenced by changes in the diversity and composition of the local plant community (Waser et al. 1996; Herrera et al. 2006; Muchhala et al. 2010). Recent studies indicate that the evolution of pollinator specialization can be influenced by coexisting plant species (Sargent and Otto 2006), to some extent driven by the level of inefficiency in pollen transfer when pollinators are visiting numerous plant species (Muchhala et al. 2010). These models assume that pollinator sharing between plant species occurs though little is known regarding the determinants of pollinator sharing (Schiestl and Schlüter 2009).

**Figure 1:**
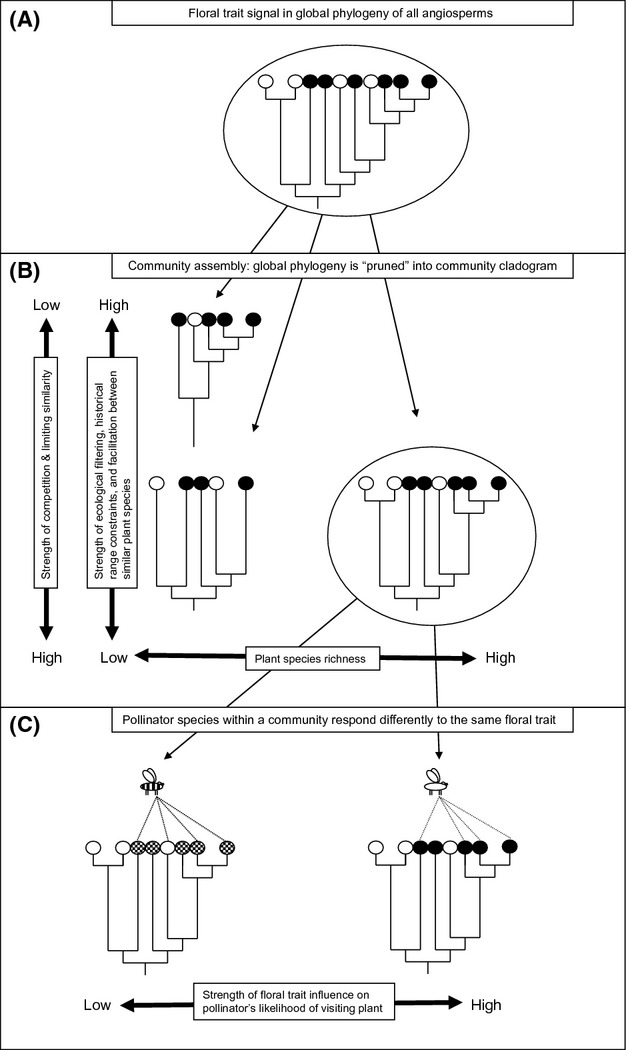
Conceptual model of mechanisms underlying the strength of phylogenetic constraints on pollination networks. (A) Phylogenetic signal for some floral traits exists in the global phylogeny of angiosperms. Open and closed circles represent the two possible states of a hypothetical binary floral trait. Close relatives are more likely to have the same trait state. (B) Community assembly “prunes” the global phylogeny into a community cladogram. Neutral, historical, and deterministic processes collectively determine the average relatedness of plants in the community cladogram, and how much phylogenetic signal of the trait will be retained. (C) Traits can vary in the strength of their effect between pollinators. In this example, all pollinators favor the trait state indicated by the darker circle. The left-hand side pollinator perceives the effect more weakly than does the pollinator on the right; therefore, the latter will experience a stronger effect of plant phylogeny.

The extent to which plants will lose pollen to other plant species will not only be influenced by how many other plant species (or individuals) are present but also by the choices pollinators make, which may be affected by the overall similarity of coflowering species. Overall similarity, in turn, is determined to some extent by shared ancestry (Sargent and Ackerly 2008). For instance, the prevalence of certain floral adaptations that act as barrier traits that restrict certain pollinators [e.g., tubular flowers or zygomorphic flowers; (Santamaria and Rodriguez-Girones 2007)] will affect the mean level of generalization in the community as well as the mean level of pollinator sharing. Pollinator guilds that can only access unrestrictive floral resources (e.g., open flowers) exploit a subset of the resources exploited compared with more “versatile” pollinators [e.g., pollinators with long tongues that allow access to nectar tubes but may also visit “open” flowers (Bastolla et al. 2009); see Fig. 1].

If “unrestrictive” plant species within a community tend to be more closely related than expected by chance (Fig. 1B), we would predict that “nonversatile pollinators”, or those that are more strongly constrained by traits, would have a high propensity for visiting closely related plants (i.e., clade specialization). This prediction can be addressed by employing phylogenetic community structure metrics (Webb 2000), examining the selected assemblage of plant species visited by each individual pollinator species compared with randomized assemblages of plant species at a site (as in Weiblen et al. 2000). When plant lineages with unrestrictive flowers experience greater pollinator sharing, selection for a decrease in the delivery of heterospecific pollen (either via reducing phenological overlap or pollinator specialization) could occur. Alternatively, if we expect that competition structures assemblages (Colwell and Winkler 1984), we might not expect much variation in the amount of phylogenetic signal in communities (McEwen and Vamosi 2010) because floral traits associated with pollination will generally be overdispersed. As a starting point to address the conceptual model in Fig. 1, we first assess the degree to which a functional “trait” exhibits a phylogenetic signal (as in McEwen and Vamosi 2010).

Pollinators may also change their clade specialization in foraging choices as the number of plant species changes within a community. Previous studies on how the mean specialization in communities varies over large geographic areas have been equivocal with regard to the influence of species richness (Olesen and Jordano 2002; Ollerton et al. 2006; Dyer et al. 2007; Dalsgaard et al. 2011; Schleuning et al. 2012). With more choices of plants to forage upon, some studies have found that pollinators increase their breadth at the species level (Lazaro et al. 2009). However, investigations that use appropriate randomization techniques find that pollinators visit fewer species than expected as plant species richness increases (Schleuning et al. 2012). When trying to address what contexts encourage clade specialization of pollinators, we thus need to examine the effects of plant species richness (Vazquez et al. 2009).

Similarly, changes in specialization at a network level could be associated with changes in the assemblages of pollinator species themselves (e.g., higher pollinator species richness, or higher proportions of pollinators with specialized preferences, such as oil-collecting bees) or because individual pollinator species have altered their level of specialization in certain contexts. Network studies have found that pollinator specialization depends not only on the plant resources on offer, but also on how many pollinator species are competing for these same resources. For example, when there are more pollinator species than plant species (e.g., network asymmetry is high), pollinators will trivially show a lower degree of specialization measured as mean degree (Bluthgen et al. 2007). However, pollinators may also change their specialization as measured using phylogenetic clustering. As pollinators can compete for floral resources (Hart and Eckhart 2010), they may alter their behavior under competition (Lazaro et al. 2010). This suggests that network asymmetry has the potential to lead to a general loss of specialization when pollinators are competing for resources. However, the extent to which network asymmetry affects community-wide patterns of specialization has rarely been assessed (but see Bluthgen et al. 2007).

To investigate how factors such as competing species and changing foraging choices alter the amount of clade specialization in visited plant species (and, by extension, the degree of pollinator sharing between relatives), we require (1) the underlying phylogenetic clustering of plant traits in communities, (2) measurement of how strongly these traits determine pollinator choice, and (3) whether the effect of these traits is dependent on plant and pollinator species richness (Fig. 1). Within this framework, we used data from 29 previously published plant–pollinator networks to examine the following predictions on how species richness and phylogenetic constraints modulate community and network structure: (1) Pollinators will generally exhibit clade specialization such that they visit plants that are more related than expected by chance; (2) clade specialization is caused in part by barrier traits, such that clade specialization will be stronger for less versatile pollinators; (3) ecological context, in terms of plant species richness and asymmetry, affects the level of species specialization and clade specialization; and finally (4) pollinator specialization is affected by pollinators altering their foraging depending on the ecological context. We test the idea that individual pollinator species alter their foraging depending on changes in the plant community using 29 previously published plant–pollinator networks, finding 44 pollinator species that were common to at least three plant–pollinator networks.

## Methods

### Datasets and phylogenies

We conducted a literature search to find community plant–pollinator interaction datasets. Datasets were used if they attempted to record all pollinator taxa visiting at least the dominant plants present in a defined community at the morphospecies or species level, resulting in 29 usable datasets (see Table 1). An initial search for datasets was performed in Web of Science (http://www.webofknowledge.com) using the search terms ((pollinat* OR ((plant* OR flower* OR floral) AND (insect*. OR visitor* or animal*)) AND (network* OR web* OR interact* OR communit*)), and by consulting the NCEAS Interaction Web Database (http://www.nceas.ucsb.edu/interactionweb/), a repository of interaction matrices hosted by National Center for Ecological Analysis and Synthesis, at the University of California, Santa Barbara, USA. A further search was made of the references within the initial papers found.

**Table 1:** Datasets used and their attributes

Dataset code	Reference of dataset source	Latitude	Plant SR	NV Poll SR	V Poll SR	Source	Spatial Scale	Temporal Scale
A1	Arroyo et al. (1982; subandean scrub)	33°17′S	69	18	31	NCEAS	500 m	6 months: October 1980–March 1981
A2	Arroyo et al. (1982; cushion zone)	33°17′S	34	11	19	NCEAS	500 m	6 months: October 1980–March 1981
A3	Arroyo et al. (1982; subnivean tussock)	33°17′S	26	3	7	NCEAS	500 m	6 months: October 1980–March 1981
BA	Barrett and Helenurm (1987)	46°33′N	12	18	13	NCEAS	2000 m^2^	3 yrs (May–September, 1978–80)
CL	Clements and Long (1923)	38°50′N	94	31	84	NCEAS	15 ha	5 years: 1918–1923
DU	Dupont et al. (2003)	28°13′N	11	10	13	NCEAS	300 m × 400 m	1 month
EB	Elberling and Olesen (1999)	68°21′N	23	38	4	NCEAS	30 m × 50 m	3 months (May–August 1994)
HE	Herrera (1988)	37°01′N	26	28	33	LS	4 ha	14 months
IU	Inoue et al. (1990)	35°10′N	114	223	57	LS	3 km	4 years 1984–1987
IY	Inouye and Pyke (1988)	36°25′S	37	32	13	NCEAS	104 m^2^	1 year: December 1983–March 1984
K1	Kato et al. (1990)	35°20′N	91	141	49	NCEAS	ND	ND
K2	Kato et al. (1993)	35°35′N	91	108	25	LS	4 km	2 years: 1990–1991
KK	Kakutani et al. (1990)	35°02′N	113	75	48	LS	1.5 km	2 years: April–November 1985–1987
KV	Kevan (1970)	81°49′N	17	31	9	NCEAS	ND	May 25–August 6 1967
ML	Medan et al. (2002; Laguna Diamante)	34°10′S	21	15	7	NCEAS	100 m × 200 m	6 days: 14–20 January 1995
MR	Medan et al. (2002; Rio Blanco)	33°00′S	23	15	13	NCEAS	100 m × 250 m	6 days: 11–17 December 1996
MS	Mosquin and Martin (1967)	75°00′N	11	8	1	NCEAS	ND	13 days (July 19–31, 1965)
MT	Motten (1982)	36°00′N	13	6	23	NCEAS	12 km	5 years: 1977–1982; during flowering period
OA	Olesen et al. (2002; Isle d'Aigrettes)	20°25′S	14	4	6	NCEAS	26 ha	2 months (November 1998 and June 1999)
OF	Olesen et al. (2002; Flores Island)	39°20′N	10	3	4	NCEAS	25 ha	1 month (July 2000)
PE	Percival (1974)	17°55′N	42	4	16	LS	10 ha	ND
PR	Primack (1983)	43°00′S	89	53	37	LS	ND	3 years: summers of 1976–1978
RA	Ramirez and Brito (1992)	8°56′N	28	13	11	NCEAS	ND	3 years: 1983, 1984, 1989
SC	Schemske et al. (1978)	40°09′N	7	6	8	NCEAS	24 ha	3 years 1974–1976
SL	Small (1976)	45°24′N	13	52	28	LS	500 m	1973 season; 1000–1500 h
SR	Smith-Ramirez et al. (2005)	42°30′S	26	50	9	LS	ND	3 years: 1999–2002
VM	Vázquez and Simberloff (2002; Mascardi – No Cows)	41°00′S	10	8	3	NCEAS	700 m	ND
VU	Vázquez and Simberloff (2002; Quetrihue – Cows)	41°00′S	11	8	4	NCEAS	700 m	ND
YA	Yamazaki and Kato (2003)	33°24′N	98	55	37	LS	ND	ND

NV, nonversatile; V, versatile; LS, dataset obtained from literature search; ND, not described.

In order to include a larger number of studies, we included communities with binary interaction data (ignoring the quantitative component of the interaction) and created networks using such data across all communities. Plant names were verified and updated where nomenclature changes have occurred, so that designation of species to genera would reflect recent changes, following International Legume Database & Information Service (2009), the Integrated Taxonomic Information System (ITIS) (http://www.itis.gov), the Flora of China and the Flora of Nepal in eFloras (2008), and Tropicos.org (http://www.tropicos.org). Plants that were present in the community but not visited by any pollinators were removed from the few datasets where these were listed. Some datasets contained some pollinator groups that were identified to very coarse levels such that they were pooled together as a single “visiting species” (e.g., all Acari); these were also removed prior to using the datasets.

We used the Phylomatic tool (Webb and Donoghue 2005) in the software package Phylocom (Webb et al. 2008) to construct cladograms of the plants present in each community. We started by producing an initial master phylogeny of all plant species present across all datasets combined. Phylomatic constructs a cladogram for a list of plant species input by the user by grafting these species onto a backbone family-level tree and then removing all higher taxa from the tree that are not represented on the list; for the tree backbone, we used the Angiosperm Phylogeny Group (APG) supertree R20090303.new (Stevens 2001onwards), further resolved using other published sources. The default APG supertree used by Phylomatic is resolved only to the family level across most of the tree. We used other sources (see Appendix S1) to resolve the relationships within families, provided that these phylogenetic relationships within the family had at least 80% support as defined by the source. We then used BLADJ package of Phylocom (Webb et al. 2008) to assign branch lengths on the master phylogeny based on dated calibration points (Wikstrom et al. 2001; Hedges et al. 2006) which used nonparametric rate smoothing to assign ages to most nodes on the tree above the level of family. The BLADJ package adjusts the remaining undated nodes at equal intervals between the dated nodes. Finally, we created individual community phylogenies by “pruning” the dated master phylogeny of all species not present in each dataset. Because we extracted a cladogram from the initial master phylogeny for each plant assemblage (Figure S1), the resulting community phylogenies were not overly plagued with unresolved nodes.

### Classification of floral morphology (restrictiveness) and pollinator type (versatility)

As an extension of Faegri and van der Pijl's (1979) description of floral morphologies and floral syndromes, we categorized plants dichotomously as “restrictive” or “unrestrictive” based on whether they possessed a morphological barrier that prevents some pollinators from accessing their floral rewards (analogous to the “accessibility” binary trait used in Fontaine et al. (2006). Flowers that are gullet-shaped, flag-shaped, urn-shaped, tubular, or spurred and other flowers with obvious restrictions on accessing rewards were classified as restrictive. Flowers with readily accessible rewards not requiring specialized morphology to access, generally encompassing dish-, bowl-, bell- and funnel-shaped flowers, were classified as unrestrictive. Where flowers had combined morphologies, we used the morphological feature that corresponded to reward access (e.g., a flower that is funnel-shaped overall, but with nectar located within a tubular base would be classified as restrictive). We used many different sources for classification, including descriptions and illustrations in floras, and photographs (source references available upon request).

**Figure 2:**
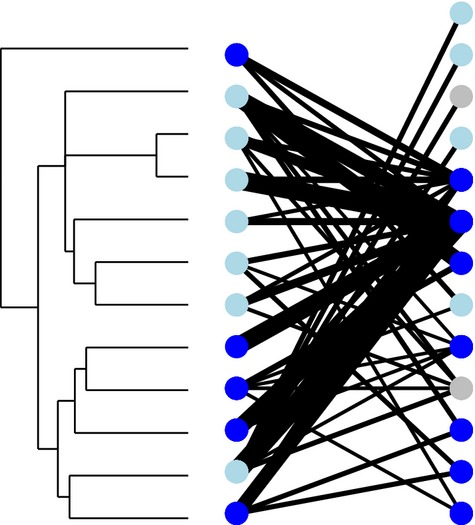
Example of community phylogeny showing floral restrictiveness (“restrictive plant species” shown in dark blue; “unrestrictive” species in light blue). The associated interaction network is shown, with corresponding blues for pollinators representing versatility: dark blue represents versatile, whereas light blue is nonversatile; gray indicates a pollinator where taxonomic data were too coarse to designate versatility. Data from Olesen et al. (2002).

Pollinators were classified as “versatile” (V) or “nonversatile” (NV) based on the general tendency within the group to have the ability to access restricted floral rewards. Bees, moths, and butterflies were classified as versatile, whereas wasps, flies, beetles, bugs (Hemiptera), and miscellaneous other pollinators (e.g., neuropterans) were classified as nonversatile. This classification of flower and pollinator types follows Faegri and van der Pijl's (1979) paradigm of “mess and soil” versus highly adapted pollinators, and their assignment of flower morphologies to these pollinator groups. Although this classification scheme is extremely simplistic, previous studies have been able to detect significant evolutionary and ecological patterns related to pollination using a simple classifications (Sargent 2004; Harmon-Threatt and Ackerly 2013). Importantly, our coarse measure of flower restrictiveness did appear to operate as an important trait determining partnerships between plants and pollinators: versatile pollinators include a higher proportion of restrictive flowered species among those that they visit than do nonversatile pollinators; the dataset median proportion of restrictive flowers for versatile and nonversatile pollinators were 0.29 and 0.13, respectively (Wilcoxon test of paired median proportions, *z* = 105.5, *P* < 0.0001, see Fig. 2). This difference between pollinator types did not change with community plant species richness (*F*_1,28_ = 0.001, *P* = 0.971, *R*^2^ < 0.01), and the proportion of unrestrictive plant species did not change with plant species richness (*F*_1,28_ = 2.014, *P* = 0.16, *R*^2^ = 0.07).

### Null model test of phylogenetic clustering of visited plants

In order to determine the degree to which the plants visited by each pollinator species were more or less closely related than expected by chance, we used a null model that assumes random visitation with respect to species identity, but maintains the other aspects of community structure, that is, the total number of plant species that a pollinator species interacts with and the total number of pollinator species a plant interacts with in the original dataset (Gotelli and Entsminger 2003). Randomization was performed using the independent swap method, using 100,000 swaps per run and 1000 runs per community in Phylocom v.4.1.

As in Weiblen et al. (2006), pollinators who visited a single plant species were excluded. Some previous studies of pollinator specialization (e.g., see Vázquez & Aizen 2004) have chosen to exclude pollinators that visit fewer than five plant taxa under the rationale that a smaller number of taxa provide an insufficient sample from which to infer a measure for the pollinator; however, this exclusion also means that a large proportion of pollinators, biased toward those that are locally rare and/or specialized, are excluded from consideration. We have included all pollinators that visited two or more taxa in this study to avoid this bias, and with the hope that although more noise is introduced into the statistical analysis, the large number of pollinators that visit few species or are rarely observed will allow more power to detect an overall difference in their mean dispersion.

### Metric of phylogenetic clustering of visited plants

We used the *comstruct* function in the software package Phylocom (Webb et al. 2008), which assesses whether the species present in a sample are phylogenetically random with respect to species available in a set; here, the plant species visited by a pollinator comprise a sample of the set of all plant species in the community. Because the common net relatedness index (NRI) metric can be biased toward overdispersion, we used a rank-based metric based on the quantiles of the randomization, which we refer to as RNRI (rank-based net relatedness index: see Appendix S2).

### Species richness of community phylogenies

Our ability to detect phylogenetic signal may depend on species richness [phylogenetic signal metrics suffer from low power when *N* < 25 (Blomberg et al. 2003)]. We calculated mean node depth for all community phylogenies as the mean of the log-transformed ages of all nodes present in the phylogeny. Where polytomies were present in the phylogeny, the node was counted toward the whole-tree average multiple times (i.e., *x*−1 times where *x* is the number of dependent branches). This procedure produces a bias toward a greater mean node depth, but this bias should not increase type I error (see Discussion). Finally, we assessed whether versatile and nonversatile pollinators altered their degree (i.e., the number of plant species visited) with species richness (i.e., whether pollinator sharing among plant species increased with plant species richness), using the “*normalized degree”* metric (Martin Gonzalez et al. 2010), which expressed the specialization of a given species in relation to what resources are on offer. For community-level metrics, we calculated the median number of plant species visited by all pollinators (as well as V and NV pollinators separately) divided by the total number of plants in the community (Waser et al. 1996). We examined whether there was substantial pseudoreplication in our communities driving patterns by examining the overall Bray–Curtis dissimilarities in their plant composition, and by considering an NMDS analysis of the species richness and number of V and NV pollinators across communities. We found pseudoreplication to be minimal (Figure S2).

### Phylogenetic signal of floral restrictiveness

To determine whether phylogenetic signal in floral restrictiveness is a plausible basis for phylogenetic clustering of plants visited by pollinators, we evaluated whether phylogenetic signal for floral restrictiveness is retained in the “pruned” phylogenies that contain only those plants present in a community and whether the strength of signal depended on plant species richness. We used the D statistic (Fritz and Purvis 2010) as a measure of phylogenetic signal of flower type (restrictive versus unrestrictive), which was calculated with the *phylo.d* function in the *caper* package of R (R Development Core Team 2010). D is based on a Brownian motion model of continuous trait evolution combined with a threshold function that allows the resulting distribution to be converted to a dichotomous trait, with the threshold chosen to yield the observed frequencies of the two states. D is therefore independent of the number of terminal taxa and trait prevalence. D scales such that a value of one indicates no phylogenetic signal, and a value of zero indicates that phylogenetic signal is the same as under a Brownian model of evolution for the threshold trait, with negative values indicating greater extremes of signal and positive values above one indicating overdispersion (Fritz and Purvis 2010). D handles polytomies well, with polytomies having little effect on D of trees with at least 70% resolution (Fritz and Purvis 2010).

### Phylogenetic clustering of visited species

Under the null hypothesis, RNRI values should be uniformly distributed with a mean of zero. If, instead, pollinators tend to visit plants that are more closely related than expected by chance, then an excess of positive RNRI values will result. Because pollinator species occurring in more than one dataset (community) cannot be considered independent data points, and because RNRI scores within datasets were not consistently normally distributed, we tested for whether RNRI values tended toward positive values based on the community-level pooled medians (i.e., the median of all pollinators within a community). We used a two-tailed *t*-test to test whether the mean community-level median RNRI differed from zero. Whenever parametric tests were used, we tested that residuals were normally distributed using the Shapiro–Wilks test. Where applicable, we tested that residuals were homoscedastic by visually examining residual plots. We used Spearman's rank correlation to test whether phylogenetic signal varies with community plant species richness. Parametric tests were not appropriate due to extreme heteroscedasticity. We tested for a relationship between plant species richness and mean phylogeny node depth using a regression of mean node depth versus log-transformed plant species richness. In phylogenetic community structure parlance, the “local assemblage” equates with the plant species visited by a given pollinator, while the “regional assemblage” is the total plant species richness in the community. We used a network-level metric analogous to (local assemblage species richness)/(regional pool species richness) by calculating (mean degree of pollinators)/(plant species richness) (or “normalized degree”). We used the calculation of network asymmetry as in Bluthgen et al. (2007) of (pollinator richness - plant richness)/(pollinator richness + plant richness). We calculated the median RNRI scores for each set of versatile and nonversatile pollinators and performed a regression of these values against ln(species richness), normalized degree, and network asymmetries of the communities to test whether RNRI depended on increased plant foraging choices, mean specialization of the pollinators themselves, or the ratio in species richness between the two, respectively.

We found 44 pollinator species that were present in ≥3 communities (20 versatile; 24 nonversatile species). We applied linear mixed effects models using the R package “lme4” to examine the effects of plant species richness, network asymmetry, and normalized degree on RNRI (random effects models, including the pollinator species identity as an additional random effect). We evaluated the effect of interactions by comparing a full model with a reduced model with a likelihood ratio test, omitting interaction terms where nonsignificant (Crawley 2007). We tested for the presence of the same relationships as in our community-level analyses described above (e.g., RNRI ∼ plant species richness * versatility). Because several pollinator species belonged to the same networks, we nested species identity within communities to avoid pseudoreplication.

## Results

### Overall presence of phylogenetic clustering

Among all the datasets, there were 4313 pollinator visitation profiles, including those where only one plant species was visited (where the same species appears in multiple networks, the records are counted as independent visitation profiles). There were 3314 pollinators species in total and 998 flowering plants species. RNRI values were calculated for 1685 pollinator records, those that visited more than one plant species. 986 (58.5%) of the pollinator species had positive RNRI values, and 699 (41.5%) had negative values. We provide the RNRI values per functional group of pollinators in Figure S3. Because some source studies specifically concentrated solely on plant–insect interactions, birds that visited more than one plant species were excluded (eight records), leaving 1680 pollinators included in the analysis. The median RNRI value pooled over all pollinators in all communities was 0.162 (mean = 0.127, suggesting moderate but consistent phylogenetic clustering (two-tailed *P* < 0.0001, *t*_1,1679_ = 8.604). Twenty-two of 29 datasets (76%) had positive median RNRI scores with an estimated mean of community median RNRI ± SE of 0.1372 ± 0.045, again indicating consistent phylogenetic clustering overall (*t*_1,28_ = 3.04, *P* = 0.005). RNRI also exhibits a positive relationship with community plant species richness (*β* = 0.10; *P* = 0.043; *R*^2^ = 0.14), suggesting that as plant communities become more species rich, pollinator clade specialization increases. Finally, RNRI was not related to network asymmetry (*β* = −0.14; *P* = 0.40; *R*^2^ = 0.03), suggesting that pollinator clade specialization is unaffected as competition increases.

### Plant species richness and phylogenetic signal of floral restrictiveness

Mean phylogeny node depth was strongly negatively correlated with community plant species richness (*F*_1,28_ = 38.5352; *P* < 0.0001; *R*^2^ = 0.59), indicating that species-poor communities consist of more distantly related species. This finding was not an artifact of the level of phylogeny resolution (with polytomies forcing longer branch lengths), as the phylogenies of more plant species-rich communities were less resolved than were the species-poorer ones (slope of number of nodes in a community tree versus community size = 0.69, significantly less than the null 1:1 line, s.e. of slope = 0.02). Twenty-one of the 29 communities (72.4%) exhibited a phylogenetic signal for floral restrictiveness (test of signal = 0; Table 2). Of these 21 communities, all but seven communities exhibited a signal that did not significantly depart from that expected under Brownian motion. As expected, communities where we could not detect phylogenetic signal had few species (*t*_1,29_ = 3.61, *P* = 0.0012).

**Table 2:** Phylogenetic signal (D) of floral restrictiveness in community phylogenies, and *P*-values corresponding to the null hypotheses of no phylogenetic signal and Brownian structure. See text for details

Dataset code	D (estimate) of floral restrictiveness	*P* (signal) (uncorrected)	*P* (Brownian structure) (uncorrected)
A1	−0.57	<0.001	0.94
A2	−0.56	<0.001	0.85
A3	−0.43	0.04	0.78
BA	3.63	0.92	0.03
CL	−0.28	<0.001	0.83
DU	0.71	0.36	0.26
EB	0.44	0.24	0.36
HE	−0.23	<0.001	0.61
IU	−0.60	<0.001	0.96
IY	−0.11	0.02	0.65
K1	−0.50	<0.001	0.93
K2	−0.33	<0.001	0.77
KK	−0.14	<0.001	0.67
KV	−2.08	0.01	0.78
ML	−3.26	<0.001	>0.99
MR	−1.40	0.03	0.94
MS	−6.79	0.03	0.89
MT	0.99	0.42	0.41
OA	−1.69	0.06	0.88
OF	−0.07	0.29	0.49
PE	0.18	<0.001	0.40
PR	0.09	<0.001	0.42
RA	−0.03	0.04	0.59
SC	−3.36	0.04	0.85
SL	−1.22	<0.001	0.84
SR	−0.57	<0.001	0.80
VM	0.82	0.44	0.44
VU	3.76	0.71	0.21
YA	−0.16	<0.001	0.66

### Effect of traits, plant species richness, and pollinator behavior on phylogenetic clustering of visited plants

**Figure 3:**
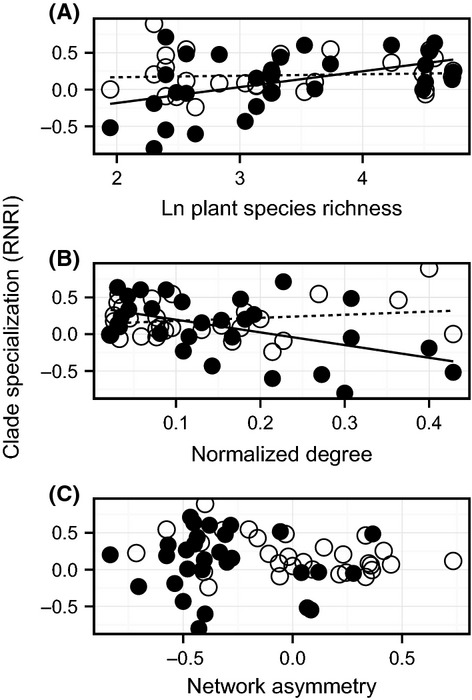
RNRI for versatile (V, solid symbols) and nonversatile (NV, open symbols) pollinators. Versatile pollinators tend to visit related plant species as plant species richness increases (A), and as normalized degree decreases (B), but are not affected by network asymmetry (C). Nonversatile pollinators tend to visit more related plant species overall but do not change their level of clade specialization with plant species richness, normalized degree, or network asymmetry. Trendlines are included to help visualize contrasting relationships, but only the solid lines between RNRI versus plant species richness and normalized degree are significant.

Median community RNRI of versatile and nonversatile pollinators differed: the nonversatile pollinators showed phylogenetic clustering (*t*_1,28_ = 4.1227, two-tailed *P* = 0.0003), while the versatile pollinators did not (*t*_1,28_ = 1.376, two-tailed *P* = 0.179). Median community RNRI of nonversatile pollinators did not vary substantially with plant species richness (*F*_1,29_ = 0.13, *P* = 0.721; Fig. 3). In contrast, clustering of plants visited by versatile pollinators increases with plant community richness, being completely absent in the smaller communities, but becoming similar to that of nonversatile pollinators in the larger plant communities (*F*_1,29_ = 7.55, *P* = 0.011; Fig. 3).

At the community level, some pollinators increased niche breadth (in terms of the sheer number of plant species visited) with increases in plant species richness as expected, but the median degree of pollinators remained unchanged (*F*_1,29_ = 1.02, *P* = 0.32). Thus, pollinators actually visit a smaller proportion of the plant species on offer as plant species richness increases (*F*_1,29_ = 96.40; *P* < 0.0001). In concert with this greater realized selectivity, we observe that clade specialization is higher in communities where the average number of plant species visited is lower (*F*_1,29_ = 4.03; *P* = 0.05). This pattern was largely due to changes in the phylogenetic clustering exhibited by versatile pollinators (*F*_1,29_ = 8.03; *P* = 0.008, Fig. 3) not due to any changes in nonversatile pollinators (*F*_1,29_ = 1.00; *P* = 0.32, Fig. 3). Because normalized degree and plant species richness exhibit such a high degree of colinearity, however, it will be hard to tease the effects of a greater number of choices in resources from innate preferences of the pollinators themselves apart.

Community-averaged clade specialization (median RNRI) did not increase with increased network asymmetry overall (*F*_1,29_ = 0.72, *P* = 0.40), or within pollinator type (versatile pollinators: *F*_1,29_ = 0.12, *P* = 0.73; nonversatile pollinators: *F*_1,29_ = 2.51, *P* = 0.12). Plant species richness and network asymmetry appear to vary independently from one another; in other words, network asymmetry does not simply increase as plant species richness decreases (in fact, the two variables showed no relationship; Spearman's *ρ* = −0.03, *P* = 0.87).

When examining pollinators that were observed in ≥3 networks, we find that there was a positive effect of plant species richness (*β* = 0.27 ± 0.10, *t* = 2.68, *n* = 111, *P* = 0.008) and versatility (*β* = 1.50 ± 0.60, *t* = 2.51, *n* = 42, *P* = 0.016) on RNRI, yet a significant interaction in the opposite direction as that of all communities pooled; that is, nonversatile pollinators increase their clade specialization when in species-rich communities, but versatile pollinators remain unchanged (*β* = −0.34 ± 0.14, *t* = −2.51, *n* = 111, *P* = 0.014; Figure S4). When we examined models with other predictor variables, we found that neither network asymmetry (*β* = −0.20 ± 0.20, *t* = −1.00, *n* = 126, *P* = 0.32) nor normalized degree (*β* = 0.33 ± 0.31, *t* = 0.42, *n* = 126, *P* = 0.67) had any effect on changes in clade specialization behavior within species, indicating that individual pollinator species do not greatly change their behavior under different ecological conditions. However and notably, 43% of the common pollinators were generalists (with negative RNRI values on average), against an expectation of just 6% across all our pollinators.

## Discussion

Some recent studies (Rezende et al. 2007; Vazquez et al. 2009; Gomez et al. 2010) claim that phylogenetic constraints are generally weak in pollination networks. However, our results indicate that phylogenetic constraints may indeed be present when important traits have a phylogenetic signal. We found that, overall, the pollinators visit assemblages of plant species that are more closely related than expected by chance, even when considered in the aggregate at the community level (i.e., the median RNRI for pollinators indicates phylogenetic clustering). While our metrics differ from that of Rezende et al. (2007), who found significant similarity in pollinator identity among closely related plants in only a minority of their datasets, we note that 24 of their 33 datasets (73%) had a positive correlation coefficient between phylogenetic relatedness of plants and similarity in the identities of the pollinators that visited them (*P* < 0.01, binomial test; 73% vs. 66% of datasets used in this study). This lends further support to the idea that phylogenetic constraints do in fact contribute to the structure of pollination networks. Our results indicate that species and clade specialization were dependent of several factors, including floral traits, plant species richness, and the overall specialization of constituent pollinators. However, pollinator specialization appeared to be unaffected by the number of other pollinator species present, with network asymmetry having little influence on the strength of clade specialization.

### The effects of plant species richness and phylogenetic signal of floral restrictiveness

We found strong support for the idea that communities with higher plant species richness would be composed of plants that are more closely related to each other [mean phylogenetic node depth was very strongly negatively correlated with richness (*R*^2^ = 0.59)]. Species-poor communities generally consist of assemblages of distantly related species where the phylogenetic signal of floral restrictiveness (and presumably other traits important in plant–pollinator linkage) has been lost. Despite several studies having shown that phylogenetic resolution and taxonomic scale can influence the ability to detect phylogenetic signal in important traits (Cavender-Bares et al. 2004), a phylogenetic signal for floral restrictiveness was present in most datasets, with the exception of six communities with fewer than 25 species (i.e., below the threshold at which D loses power to detect phylogenetic signal as seen in Fritz and Purvis 2010). While a less appropriate metric to use for binary traits, we note that qualitatively similar results were obtained when we use lambda to measure phylogenetic signal (Pagel 1997; data not shown).

### Effect of pollinators on specialization of visited plants

As in previous studies (Santamaria and Rodriguez-Girones 2007), barrier traits do structure the plant–pollinator interactions observed in networks and partly drive pollinator specialization. It has often been thought that unversatile pollinators such as flies were opportunistic generalists (Kearns 2001), yet we find that pollinators without specialized mouthparts are actually constrained to visit a smaller subset of species within the community compared with bees and lepidopterans with mouthparts adapted for accessing nectar. Furthermore, the lack of versatility (e.g., exhibited by most flies) constrains them to “unrestrictive” plant clades, thus making the assemblages of plant species visited by nonversatile pollinators to be phylogenetically clustered. This indicates that clade specialization is often imposed by the availability of floral resources (and their relatedness) that pollinators are physically able to exploit. Versatile pollinators, on the other hand, may relax their floral trait specificity (and thereby the phylogenetic clustering of visited species) in communities with low plant richness, yet exhibit preferences for restrictive species (and thus clade specialization) when they are available. We were able to detect these patterns even with our relatively coarse metrics of pollinator “versatility” and plant “restrictiveness”. More fine-scaled studies in a smaller number of networks could potentially put quantitative estimates of the restrictiveness or versatility of the particular species (e.g., short, tubular flowers may actually be accessible by unversatile pollinators).

Studies have found that the extent of relatedness in assemblages depends in part on the ratio of the size of the assemblage pool to the regional pool (Kraft et al. 2007; Hoiss et al. 2012) and this is analogous to the relationship of normalized degree versus plant species richness in our study. Generally, we observed that communities harboring pollinators that visit fewer of the available plant species exhibit increased community-level clade specialization. It is difficult to translate these patterns into direct effects of sharing pollinators from the plant's perspective. However, this scenario indicates that plant species that are visited by specialized pollinators may tend to share pollinators with their close relatives, a process which may in turn instigate character displacement, and potentially speciation. Whether visitation by specialist pollinators is associated with greater diversification rates is not often examined, yet is certainly a posited mechanism for high diversification rates (Fulton and Hodges 1999).

### Pollinator specialization with changing ecological context

Plant and pollinator species richness do not vary in concert (Potts et al. 2003). This allows for an independent effect of both of these variables on, for example, clade specialization. We posit that increases in plant species richness increase the foraging choices for pollinators, while changes in network asymmetry provide a coarse surrogate for the amount of competition for a given unit of resource. We find, however, that pollinators remained unchanged in their level of specialization (both in terms of normalized degree and RNRI) with increasing plant community size (resource pool) and with increasing network asymmetry (our surrogate for interspecific competition for available resources). Thus, specialist pollinators are choosy in the number and composition of plants that they visit, but this choosiness is not a result of being relegated to use a smaller subset of available resources by competition from other pollinator species.

We note that our study did not include visitation abundance data in order to include as many networks as possible. Floral constancy of pollinators (Chittka 1992) would likely mean that including frequency data would simply increase the levels of specialization in our results, rendering our observed patterns conservative. Our analysis of individual pollinator species in different contexts contrasts with previous studies on how pollinators alter niche breadth with increasingly diverse resources (Lazaro et al. 2009) and/or competition for these resources (Fontaine et al. 2006). For instance, bees specialized when other pollinating species are introduced into the competitive arena in an experimental study (Fontaine et al. 2006), while our study finds little to suggest that pollinators alter their foraging in terms of the phylogenetically clustering of the visited plants species in response to the ratio of pollinator versus plant species richness. However, previous studies have not taken phylogeny into account and have simply examined the number of species visited. Further investigation will be needed to tease apart the confounds of changing resources, access, and competition to these resources when abundance of all the species is incorporated into the different ways of measuring specialization.

## Conclusion

The effect of plant species richness on the relatedness of plants visited by versatile pollinators reinforces the idea that the strength of phylogenetic constraints measured within a pollination network can also depend on extrinsic ecological factors. Overall, our findings suggest that conditions that favor specialists (either because of constraints or due to their prevalence in species-rich environments) contribute to the importance of phylogeny in plant–pollinator networks. Intriguingly, this specialization could increase pollinator sharing between close relatives (Padron et al. 2009) and so would be expected to select for further pollinator specialization (Muchhala et al. 2010).

## Supporting Information

**Figure S1.** The phylogenic hypotheses generated for the plant communities used in this analysis.

**Figure S2.** Communities used in these analyses are global in scope (see Table 1).

**Figure S3.** A breakdown of RNRI for the different orders examined in this study (grouped by community).

**Appendix S1.** Additional information for phylogeny resolution below the family level. **Appendix S2.** Information on the generation of the RNRI metric. **Appendix S3.** 44 pollinator species found in >3 communities, showing the heterogeneity in specialization along spatial gradients.
