# Supplementary figures and images for: Pollinators visit related plant species across 29 plant–pollinator networks
Source: Ecol Evol. 2014 May 10;4(12):2303–15. doi: 10.1002/ece3.1051 (PMC4203281; doi:10.1002/ece3.1051)

SuppFig1: The phylogenic hypotheses generated for the plant communities used in this analysis.


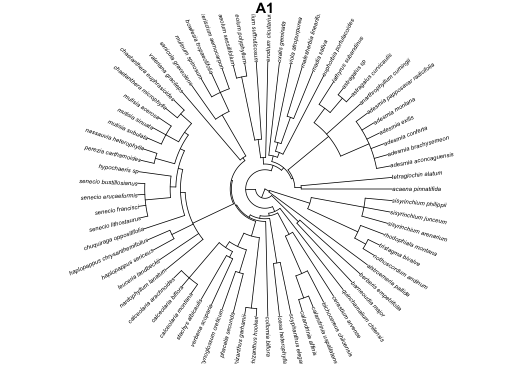

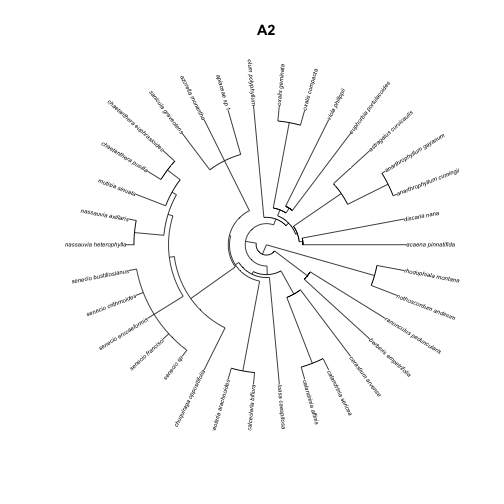

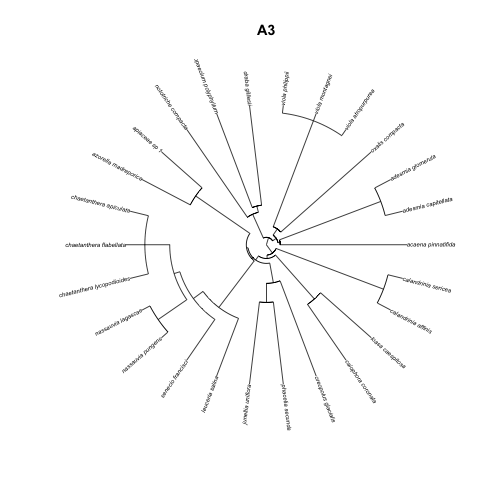

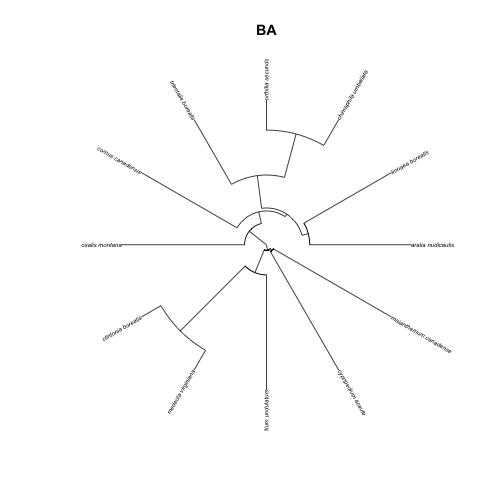

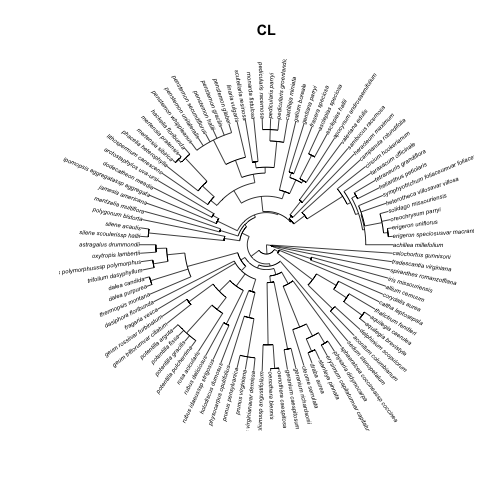

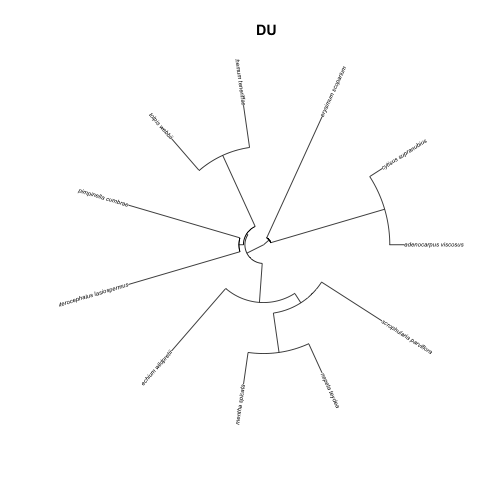

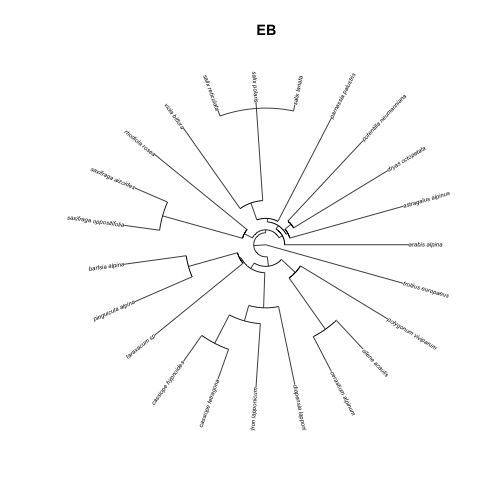

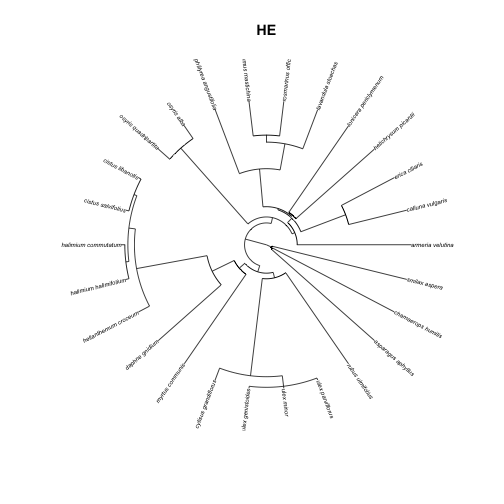

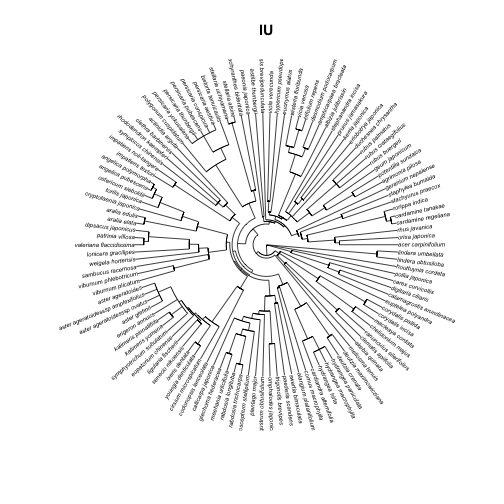

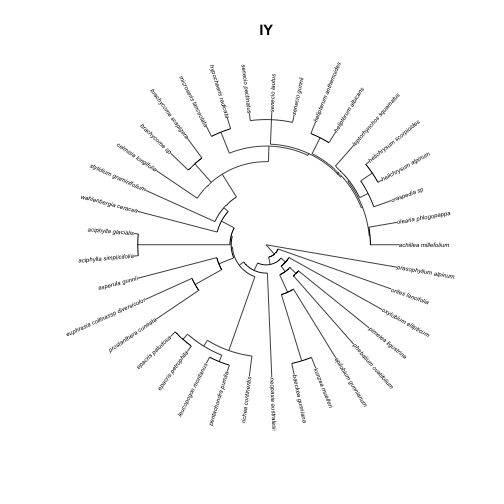

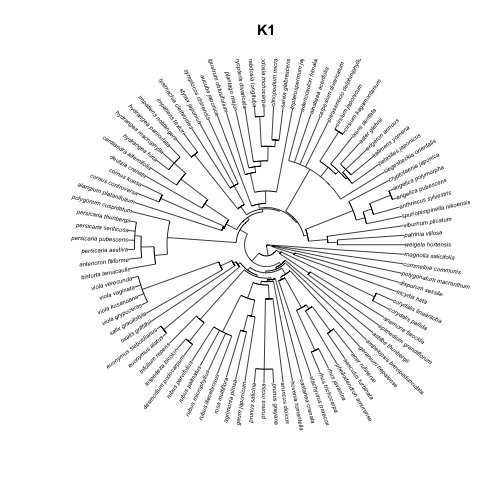

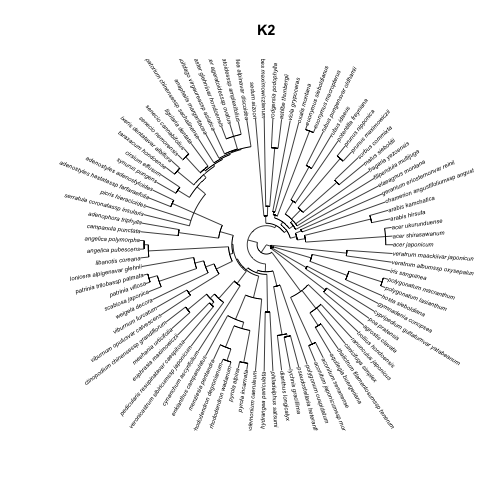

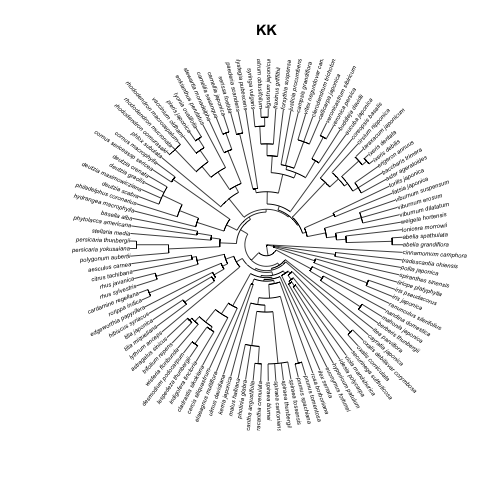

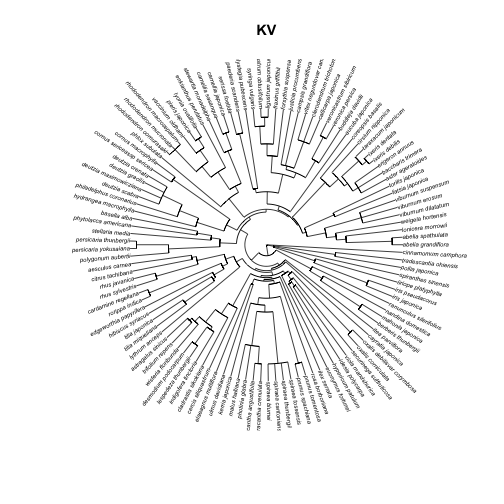

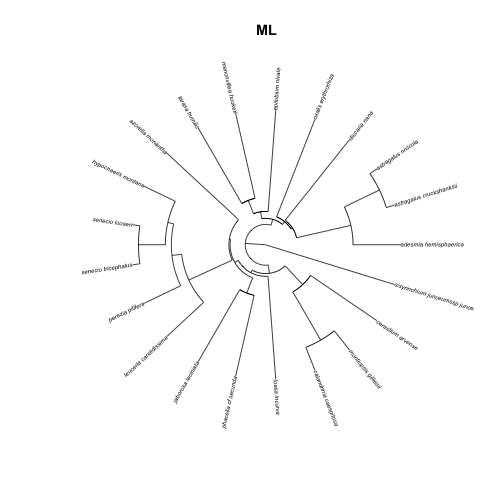

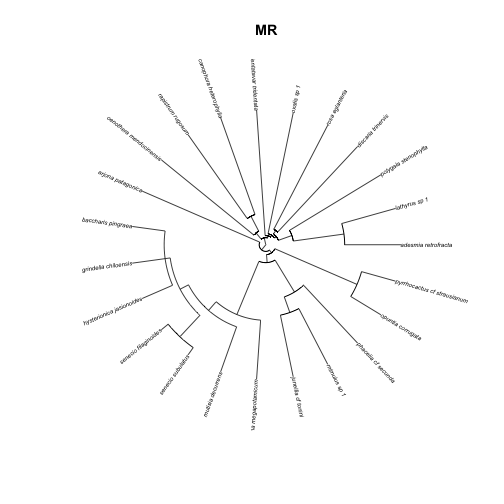

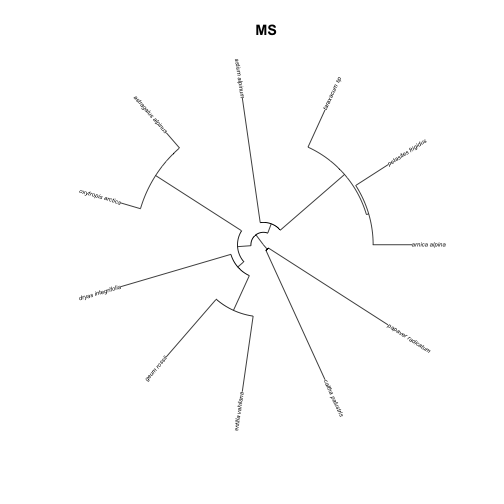

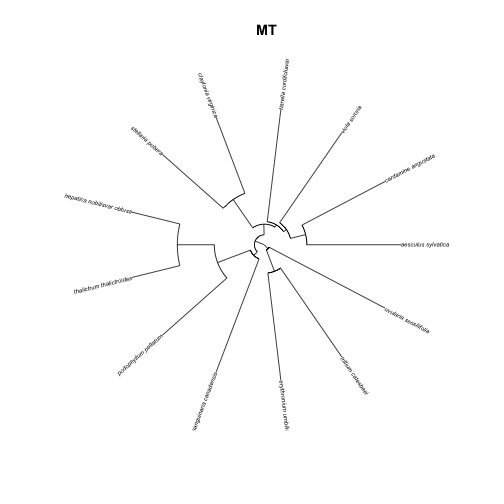

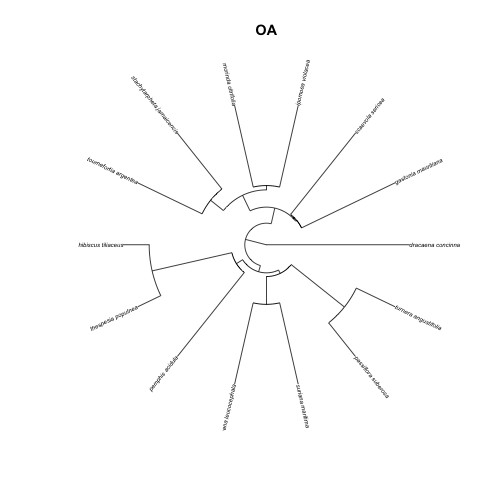

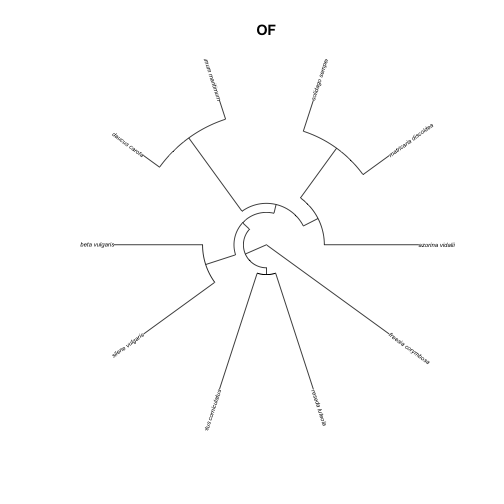

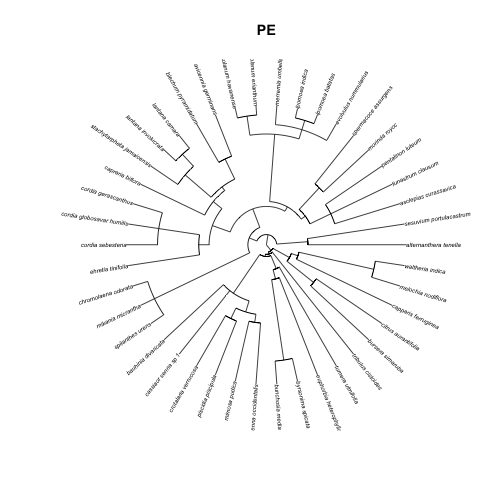

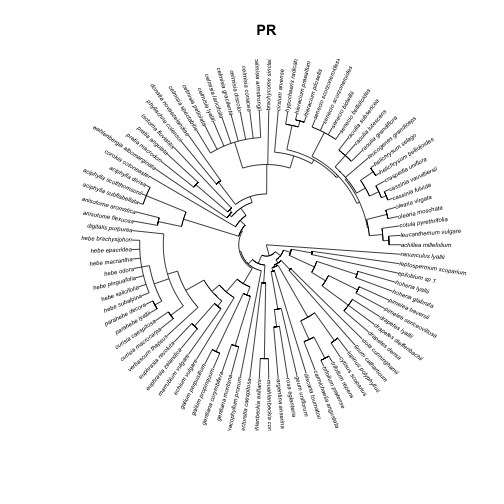

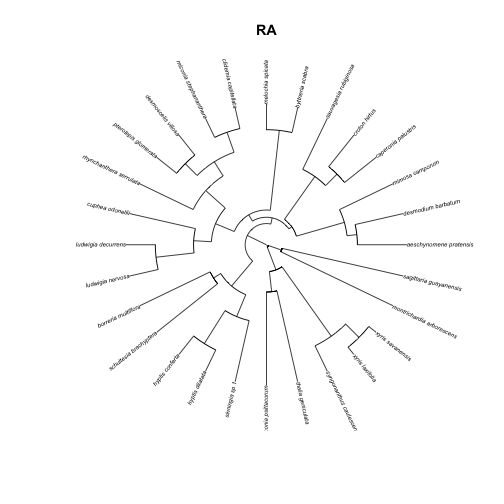

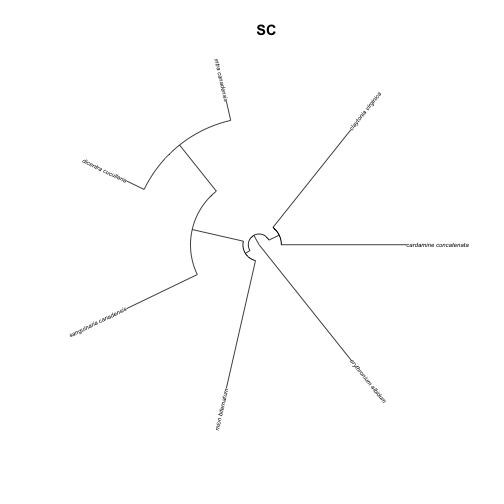


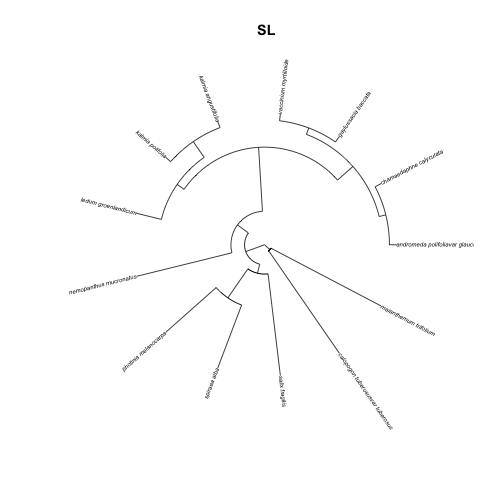

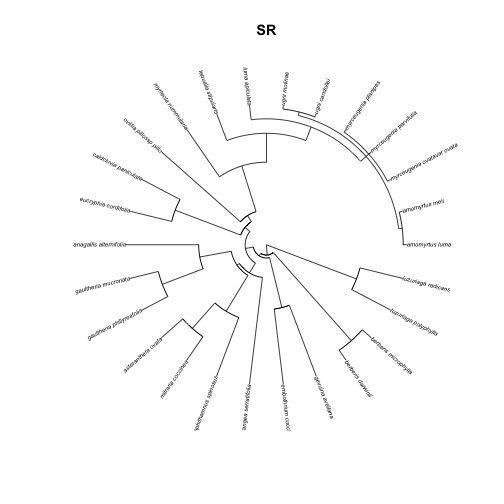

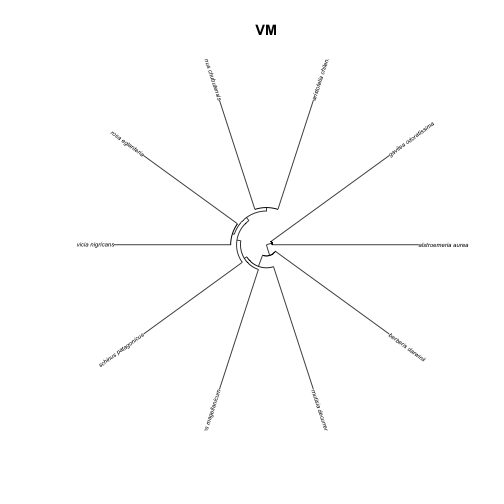

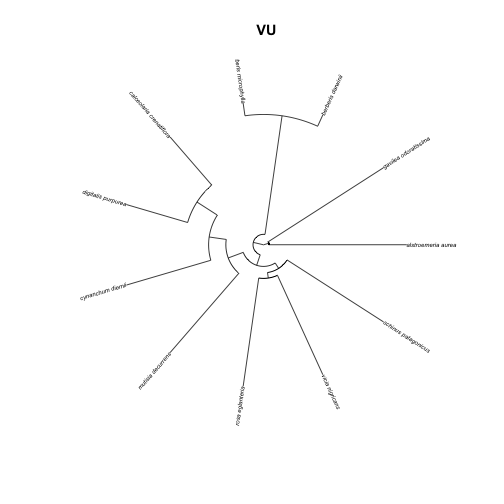

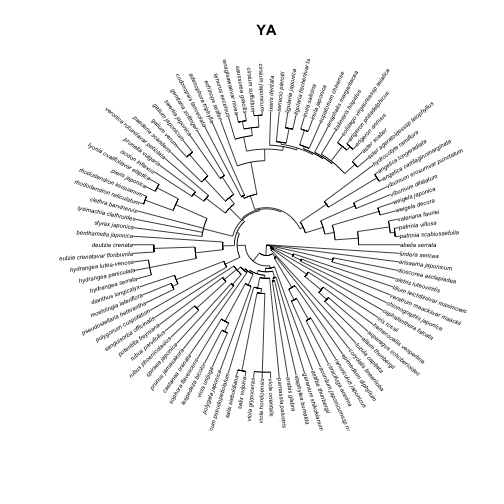

Supplement: Supplementary file 1 — Figure S1. The phylogenic hypotheses generated for the plant communities used in this analysis. [file ece30004-2303-sd1.docx]
