# Supplementary material for: Pollinators visit related plant species across 29 plant–pollinator networks
Source: Ecol Evol. 2014 May 10;4(12):2303–15. doi: 10.1002/ece3.1051 (PMC4203281; doi:10.1002/ece3.1051)

SuppFig2: Communities used in these analyses are global in scope (see Table 1). Because the majority of sites are so distant from one another, a cluster dendrogram of dissimilarities indicate that the communities are reasonably equidistant in terms of plant species composition (A). As well, non-metric multidimensional scaling also indicates little clustering in phylogenetic variables (D, nodedepth, RNRI) and number of V and NV pollinators (B). Because of these differences between communities, clustering of nearby sites (e.g., VU, VM) are not consistent drivers of the patterns seen here (C,D).

A.


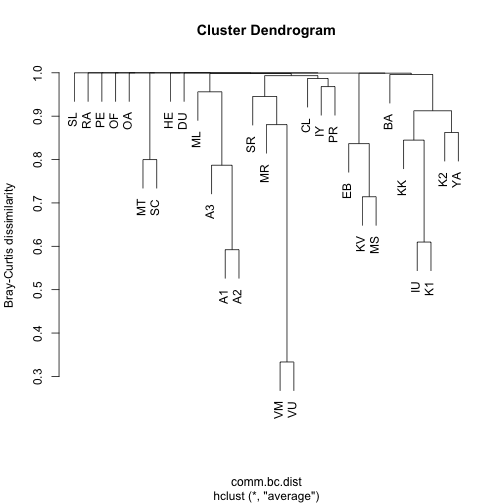


B.
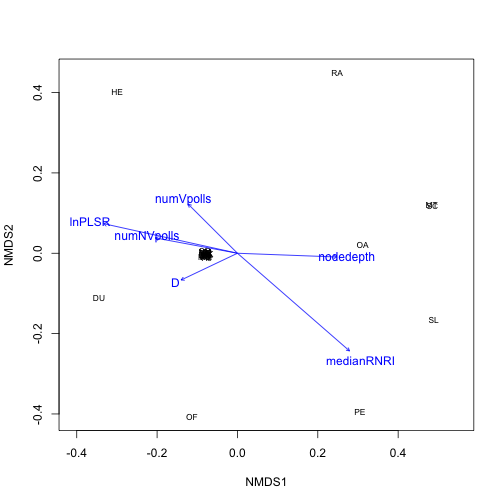


C.


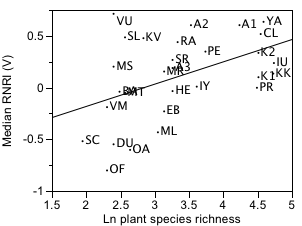


D.


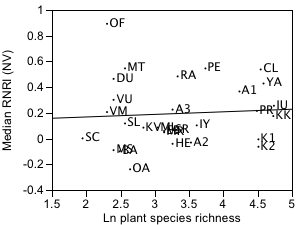

Supplement: Supplementary file 2 — Figure S2. Communities used in these analyses are global in scope (see Table 1). [file ece30004-2303-sd2.docx]
