# Supplementary material for: Pollinators visit related plant species across 29 plant–pollinator networks
Source: Ecol Evol. 2014 May 10;4(12):2303–15. doi: 10.1002/ece3.1051 (PMC4203281; doi:10.1002/ece3.1051)

SuppFig3: A breakdown of RNRI for the different orders examined in this study (grouped by community). Due to the importance of bees as a functional group, we separated them from other hymenopterans such as wasps and ants (OtherHym). “Other” includes thrips, Hemiptera, Collembola, Acari, etc., which are rarely observed as visitors and effectiveness as pollinators is unknown. All pooled, functional groups of pollinators did not show strikingly different patterns of RNRI (F-statistic: 1.991 on 6 and 3519 DF, p-value: 0.06349)


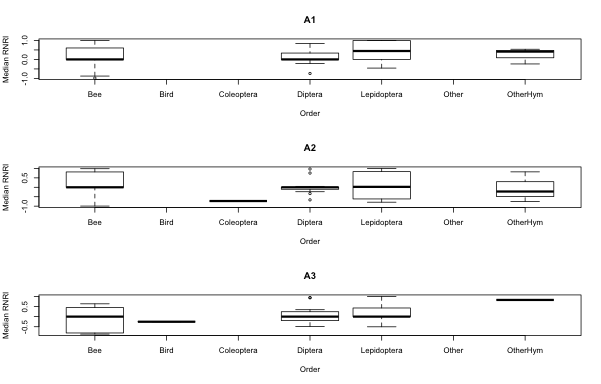


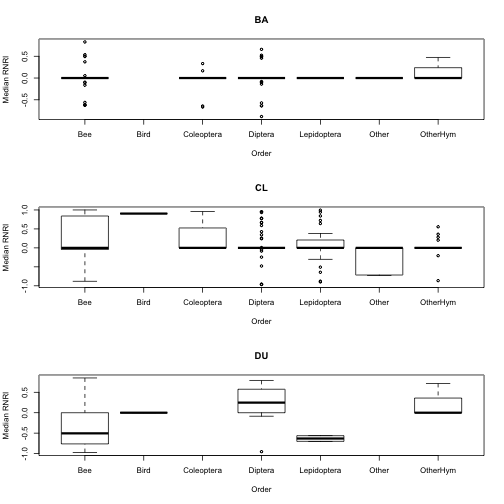


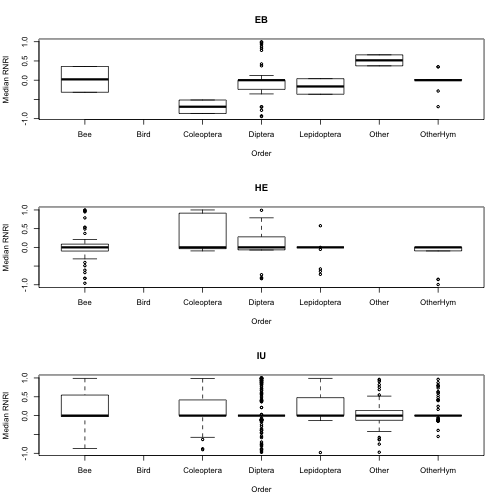


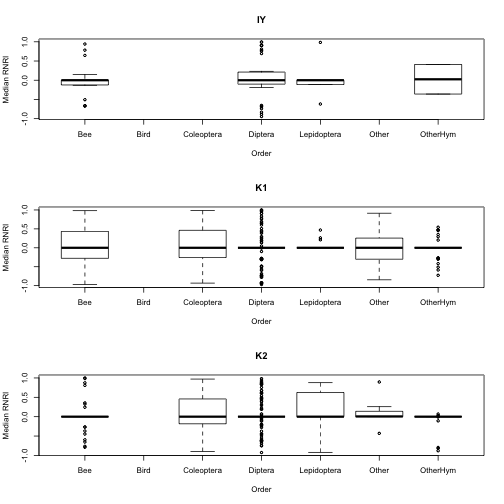


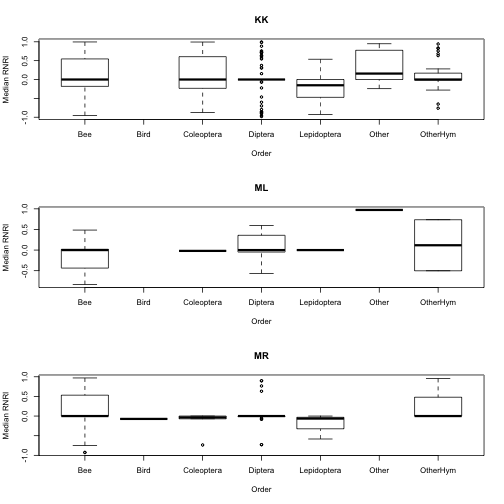


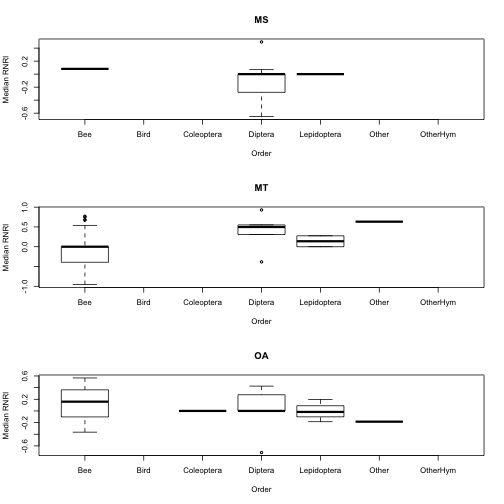


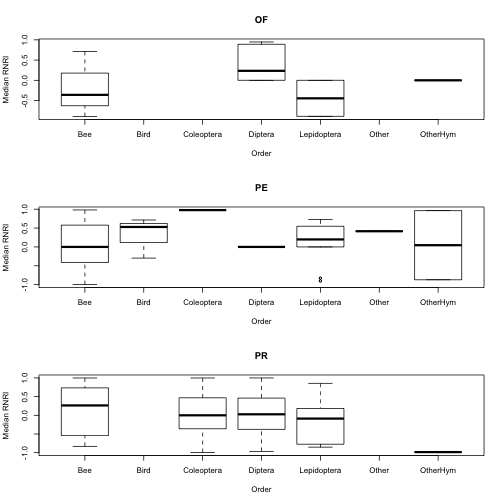


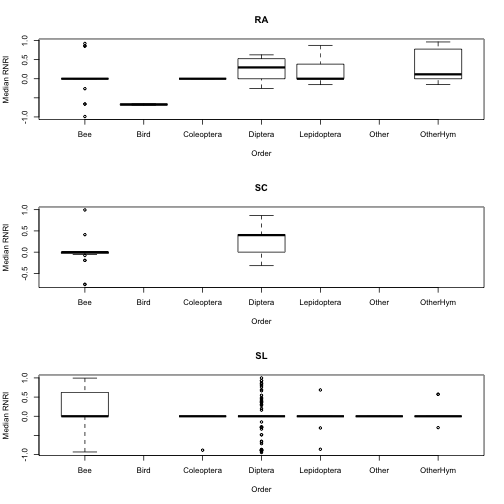


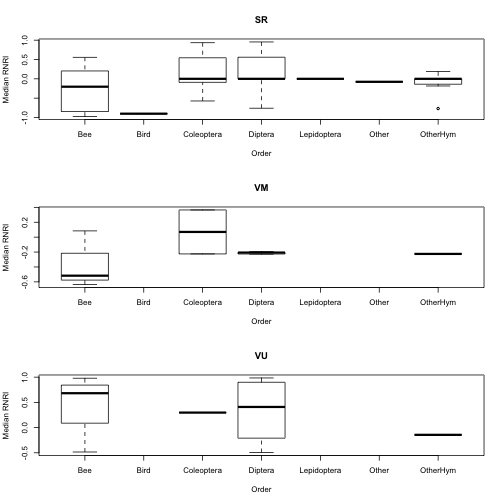


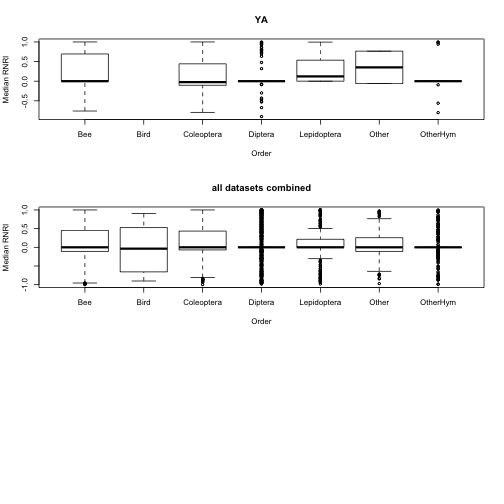


SuppFig4: Analysis of RNRI for pollinator species observed in >3 networks. We find that nonversatile pollinators (solid line) increase in clade specialization while versatile pollinators (dotted line) do not.


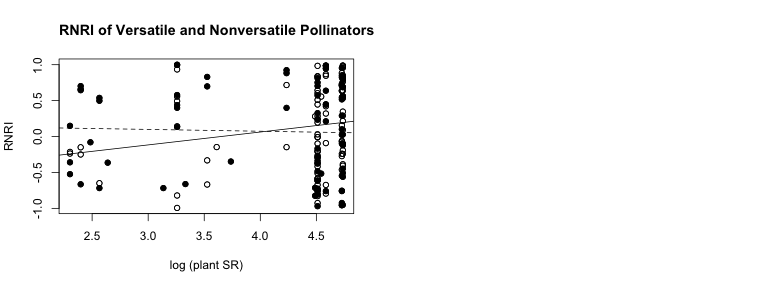

Supplement: Supplementary file 3 — Figure S3. A breakdown of RNRI for the different orders examined in this study (grouped by community). [file ece30004-2303-sd3.docx]
