# Supplementary material for: Pollinators visit related plant species across 29 plant–pollinator networks
Source: Ecol Evol. 2014 May 10;4(12):2303–15. doi: 10.1002/ece3.1051 (PMC4203281; doi:10.1002/ece3.1051)
Supplement: Supplementary file 4 [file ece30004-2303-sd4.docx]

Appendix S1:

The following families were considered for higher resolution using sources other than the APG tree:

• Fabaceae: The APG tree was resolved to the level of genus for many genera; genera occurring in datasets but not included in APG tree were assigned to the lowest possible taxon in the backbone tree between the family and generic level using LegumeWeb (International Legume Database & Information Service 2009) and The Pea Key (Australian Pea-Flowered Legume Research Group 2009).

• Rosaceae, Malvaceae, Ericaceae, Papaveraceae, Ranunculaceae and Orchidaceae: Phylogenetic relationships within the family Rosaceae were taken from the APG website (Stevens 2001 and onwards; accessed June 10-11, 2009) and species were assigned to taxa according to the Germplasm Research Information Network (GRIN) website (USDA 2009, accessed June 10-11, 2009).

• Lamiaceae and Scrophulariacaeae: Phylogenetic relations within these families are currently too poorly resolved to allow resolution of the tree below the family level (Stevens 2001 and onwards).

• Asteraceae: Phylogenetic relationships to the level of tribe were taken from Funk et al. 2005. Species in my datasets were assigned to tribes using the NCBI taxonomy browser (National Center for Biotechnology Information, U.S. National Library of Medicine 2009; retrieved June 11,2009).

• Polygonaceae and Myrtaceae: according to the GRIN website (USDA 2009, accessed June 11, 2009), all genera within these families that occurred within my datasets were within a single subfamily and no resolution was available below the level of subfamily from the APG website (Stevens 2001 and onwards).

Appendix S2

The Net-Relatedness Index (NRI) that Phylocom computes for each sample can be biased, because it assumes a normal distribution of mean phylogenetic distances (MPD) between members of the sample under the null model, while the distribution is expected to be right-skewed, biasing NRI towards underdispersion. MPD distributions derived from repeated randomizations of the community should tend to be right-skewed for two reasons: first, the distribution is bounded at zero (i.e., there is no such thing as a negative phylogenetic distance); and secondly, any amount of tree imbalance will tend to increase the relative proportion of smaller phylogenetic distances. Therefore, the median MPD from a series of randomizations (representing the midpoint of the probability distribution) will tend be to the right of the mean, so that NRI is biased towards finding phylogenetic clustering. The use of standard deviation in calculating NRI produces further problems with the interpretation of this index.

To address these problems we used a nonparametric metric calculated from the phylocom output, Rank-based Net-Relatedness Index (RNRI), which eliminates this problem of bias because it is based on the actual distribution of MPDrnd values.

The original NRI is calculated as (MPDobs-MPDrnd)/SD(MPDrnd), where

MPDobs is the mean pairwise phylogenetic distance between all species present in the observed sample from the dataset, MPDrnd is the mean of the MPD values calculated for corresponding samples generated under the null-model randomizations of the dataset, and SD(MPDrnd) is the standard deviation of these randomly generated MPD values (Webb et al. 2008b). We used the proportion of the randomly generated MPD values that lie between the observed value and the median of the randomly generated MPD values, multiplied by two, and with sign assigned such that RNRI is positive when MPDobs < median(MPDrnd) and negative when MPDobs > median(MPDrnd). RNRI ranges from -1 (maximally phylogenetically overdispersed) to +1 (maximally phylogenetically clustered), with 0 corresponding to no phylogenetic signal. In order to calculate RNRI, we used the rank information output by Phylocom (MPD.rankLow and MPD.rankHi). RNRI is equal to ((MPD.rankLow/runs) – (MPD.rankHi/runs)), where MPD.rankLow is the number of runs where MPDobs is lower than or equal to the MPD from the randomization run; and MPD.rankHi is the number of runs where MPDobs is higher than or equal to the MPD from the randomization run.

Appendix S3: 44 pollinator species found in >3 communities, showing the heterogeneity in specialization along spatial gradients.

| Species Name | V | Site | N_plant_ | N_poll_ | RNRI | Deg |
| --- | --- | --- | --- | --- | --- | --- |
| apis_mellifera | 1 | CL | 94 | 115 | -0.516 | 22 |
| apis_mellifera | 1 | DU | 11 | 23 | -0.665 | 6 |
| apis_mellifera | 1 | HE | 26 | 61 | 1 | 14 |
| apis_mellifera | 1 | K1 | 91 | 190 | -0.337 | 5 |
| apis_mellifera | 1 | KK | 113 | 123 | -0.754 | 15 |
| apis_mellifera | 1 | MR | 23 | 28 | -0.718 | 2 |
| apis_mellifera | 1 | MT | 13 | 29 | -0.718 | 6 |
| apis_mellifera | 1 | OA | 14 | 10 | -0.364 | 12 |
| apis_mellifera | 1 | OF | 10 | 7 | -0.359 | 3 |
| apis_mellifera | 1 | PE | 42 | 20 | -0.348 | 20 |
| apis_mellifera | 1 | PR | 89 | 90 | -0.714 | 4 |
| apis_mellifera | 1 | RA | 28 | 24 | -0.662 | 8 |
| apis_mellifera | 1 | SL | 13 | 80 | 0.538 | 5 |
| apis_mellifera | 1 | SR | 26 | 59 | 0.4 | 18 |
| apis_mellifera | 1 | YA | 98 | 92 | -0.76 | 11 |
| athalia_japonica | 0 | IU | 114 | 280 | 0.639 | 3 |
| athalia_japonica | 0 | K1 | 91 | 190 | -0.726 | 2 |
| athalia_japonica | 0 | KK | 113 | 123 | -0.76 | 2 |
| betasyrphus_serarius | 0 | IU | 114 | 280 | 0.686 | 5 |
| betasyrphus_serarius | 0 | K1 | 91 | 190 | -0.296 | 2 |
| betasyrphus_serarius | 0 | YA | 98 | 92 | 0.868 | 5 |
| bombus_dahlbomii | 1 | A1 | 69 | 49 | 0.882 | 7 |
| bombus_dahlbomii | 1 | A2 | 34 | 30 | 0.698 | 8 |
| bombus_dahlbomii | 1 | A3 | 26 | 10 | 0.438 | 3 |
| bombus_dahlbomii | 1 | SR | 26 | 59 | 0.134 | 21 |
| bombus_dahlbomii | 1 | VM | 10 | 11 | -0.524 | 4 |
| bombus_dahlbomii | 1 | VU | 11 | 12 | 0.702 | 7 |
| bombus_ardens | 1 | IU | 114 | 280 | 0.028 | 14 |
| bombus_ardens | 1 | K1 | 91 | 190 | -0.614 | 8 |
| bombus_ardens | 1 | K2 | 91 | 133 | -0.37 | 12 |
| bombus_ardens | 1 | KK | 113 | 123 | 0.024 | 8 |
| bombus_diversus_diversus | 1 | IU | 114 | 280 | 0.692 | 37 |
| bombus_diversus_diversus | 1 | K1 | 91 | 190 | -0.97 | 25 |
| bombus_diversus_diversus | 1 | K2 | 91 | 133 | -0.764 | 14 |
| bombus_diversus_diversus | 1 | KK | 113 | 123 | -0.955 | 2 |
| bombus_diversus_diversus | 1 | YA | 98 | 92 | 0.212 | 13 |
| bombus_honshuensis | 1 | IU | 114 | 280 | 0.816 | 9 |
| bombus_honshuensis | 1 | K1 | 91 | 190 | 0.752 | 25 |
| bombus_honshuensis | 1 | K2 | 91 | 133 | 0.326 | 20 |
| bombus_hypocrita_hypocrita | 1 | IU | 114 | 280 | -0.508 | 18 |
| bombus_hypocrita_hypocrita | 1 | K1 | 91 | 190 | -0.168 | 14 |
| bombus_hypocrita_hypocrita | 1 | K2 | 91 | 133 | 0.244 | 18 |
| bombus_hypocrita_hypocrita | 1 | KK | 113 | 123 | -0.546 | 7 |
| bombylius_major | 1 | BA | 12 | 31 | -0.08 | 2 |
| bombylius_major | 1 | IU | 114 | 280 | -0.448 | 5 |
| bombylius_major | 1 | K1 | 91 | 190 | -0.484 | 5 |
| bombylius_major | 1 | KK | 113 | 123 | 0.735 | 3 |
| bombylius_major | 1 | MT | 13 | 29 | 0.498 | 9 |
| bombylius_major | 1 | YA | 98 | 92 | 0.952 | 4 |
| carpophilus_chalybeus | 0 | IU | 114 | 280 | 0.956 | 4 |
| carpophilus_chalybeus | 0 | K1 | 91 | 190 | 0.446 | 2 |
| carpophilus_chalybeus | 0 | KK | 113 | 123 | -0.273 | 2 |
| ceratina_japonica | 1 | IU | 114 | 280 | 0.532 | 34 |
| ceratina_japonica | 1 | K1 | 91 | 190 | -0.748 | 8 |
| ceratina_japonica | 1 | KK | 113 | 123 | 0.517 | 2 |
| ceratina_japonica | 1 | YA | 98 | 92 | 0.638 | 15 |
| ceratina_megastigmata | 0 | IU | 114 | 280 | 0.564 | 22 |
| ceratina_megastigmata | 0 | K1 | 91 | 190 | 0.03 | 23 |
| ceratina_megastigmata | 0 | YA | 98 | 92 | 0.988 | 7 |
| episyrphus_balteatus | 0 | HE | 26 | 61 | -0.821 | 3 |
| episyrphus_balteatus | 0 | IU | 114 | 280 | -0.386 | 31 |
| episyrphus_balteatus | 0 | K1 | 91 | 190 | 0.834 | 6 |
| episyrphus_balteatus | 0 | KK | 113 | 123 | -0.072 | 10 |
| eristalis_tenax | 0 | DU | 11 | 23 | 0.66 | 5 |
| eristalis_tenax | 0 | HE | 26 | 61 | 0.492 | 10 |
| eristalis_tenax | 0 | IY | 37 | 45 | -0.147 | 2 |
| eristalis_tenax | 0 | K2 | 91 | 133 | 0.984 | 21 |
| eristalis_tenax | 0 | PR | 89 | 90 | 0.28 | 5 |
| eristalis_tenax | 0 | YA | 98 | 92 | 0.846 | 15 |
| eristalis_cerealis | 0 | IU | 114 | 280 | 0.972 | 19 |
| eristalis_cerealis | 0 | K1 | 91 | 190 | 0.644 | 21 |
| eristalis_cerealis | 0 | K2 | 91 | 133 | -0.007 | 22 |
| eristalis_cerealis | 0 | KK | 113 | 123 | -0.06 | 11 |
| eristalis_cerealis | 0 | YA | 98 | 92 | 0.32 | 6 |
| eurystylus_coelestialium | 0 | IU | 114 | 280 | 0.092 | 10 |
| eurystylus_coelestialium | 0 | K1 | 91 | 190 | -0.2 | 5 |
| eurystylus_coelestialium | 0 | KK | 113 | 123 | 0.851 | 3 |
| faunula_leucoglene | 1 | A1 | 69 | 49 | 0.924 | 3 |
| faunula_leucoglene | 1 | A2 | 34 | 30 | 0.83 | 5 |
| faunula_leucoglene | 1 | A3 | 26 | 10 | 0.996 | 4 |
| formica_japonica | 0 | K1 | 91 | 190 | -0.586 | 4 |
| formica_japonica | 0 | KK | 113 | 123 | 0.824 | 7 |
| formica_japonica | 0 | YA | 98 | 92 | -0.092 | 2 |
| habromyia_lipoflava | 0 | A1 | 69 | 49 | 0.717 | 2 |
| habromyia_lipoflava | 0 | A2 | 34 | 30 | -0.332 | 5 |
| habromyia_lipoflava | 0 | A3 | 26 | 10 | 0.934 | 2 |
| helophilus_virgatus | 0 | IU | 114 | 280 | 0.988 | 10 |
| helophilus_virgatus | 0 | K1 | 91 | 190 | -0.585 | 5 |
| helophilus_virgatus | 0 | K2 | 91 | 133 | 0.84 | 2 |
| helophilus_virgatus | 0 | KK | 113 | 123 | 0.714 | 5 |
| helophilus_virgatus | 0 | YA | 98 | 92 | 0.944 | 5 |
| lasioglossum_japonicum | 1 | IU | 114 | 280 | 0.56 | 8 |
| lasioglossum_japonicum | 1 | K1 | 91 | 190 | -0.287 | 2 |
| lasioglossum_japonicum | 1 | KK | 113 | 123 | 0.954 | 9 |
| lasioglossum_occidens | 1 | IU | 114 | 280 | 0.8 | 18 |
| lasioglossum_occidens | 1 | K1 | 91 | 190 | 0.572 | 8 |
| lasioglossum_occidens | 1 | KK | 113 | 123 | -0.098 | 19 |
| lasioglossum_apristum | 1 | IU | 114 | 280 | 0.852 | 38 |
| lasioglossum_apristum | 1 | K1 | 91 | 190 | 0.704 | 16 |
| lasioglossum_apristum | 1 | K2 | 91 | 133 | -0.266 | 12 |
| lasioglossum_apristum | 1 | YA | 98 | 92 | 0.449 | 2 |
| lasius_niger | 0 | HE | 26 | 61 | -0.994 | 7 |
| lasius_niger | 0 | IU | 114 | 280 | 0.808 | 6 |
| lasius_niger | 0 | K1 | 91 | 190 | -0.418 | 4 |
| lasius_niger | 0 | KK | 113 | 123 | 0.634 | 5 |
| megachile_tsurugensis | 1 | IU | 114 | 280 | -0.122 | 7 |
| megachile_tsurugensis | 1 | K1 | 91 | 190 | 0.812 | 3 |
| megachile_tsurugensis | 1 | KK | 113 | 123 | 0.29 | 6 |
| megachile_tsurugensis | 1 | YA | 98 | 92 | 0.987 | 2 |
| megaspis_zonata | 0 | IU | 114 | 280 | 0.986 | 12 |
| megaspis_zonata | 0 | K1 | 91 | 190 | 0.195 | 3 |
| megaspis_zonata | 0 | KK | 113 | 123 | 0.705 | 3 |
| melanostoma_scalare | 0 | IU | 114 | 280 | -0.952 | 26 |
| melanostoma_scalare | 0 | K1 | 91 | 190 | 0.234 | 8 |
| melanostoma_scalare | 0 | K2 | 91 | 133 | -0.926 | 7 |
| melanostoma_scalare | 0 | KK | 113 | 123 | -0.957 | 3 |
| nomada_ginran | 1 | IU | 114 | 280 | -0.561 | 5 |
| nomada_ginran | 1 | K1 | 91 | 190 | -0.804 | 3 |
| nomada_ginran | 1 | KK | 113 | 123 | 0.1 | 3 |
| nonartha_cyaneum | 0 | IU | 114 | 280 | -0.406 | 16 |
| nonartha_cyaneum | 0 | K1 | 91 | 190 | -0.673 | 4 |
| nonartha_cyaneum | 0 | KK | 113 | 123 | -0.462 | 16 |
| oedemeronia_lucidicollis | 0 | IU | 114 | 280 | -0.192 | 5 |
| oedemeronia_lucidicollis | 0 | K1 | 91 | 190 | -0.83 | 2 |
| oedemeronia_lucidicollis | 0 | YA | 98 | 92 | -0.794 | 8 |
| oxycetonia_jucunda | 0 | IU | 114 | 280 | 0.347 | 2 |
| oxycetonia_jucunda | 0 | KK | 113 | 123 | 0.64 | 14 |
| oxycetonia_jucunda | 0 | YA | 98 | 92 | 0.421 | 5 |
| philopota_nigroaenea | 0 | IU | 114 | 280 | 0.824 | 4 |
| philopota_nigroaenea | 0 | K1 | 91 | 190 | 0.284 | 2 |
| philopota_nigroaenea | 0 | KK | 113 | 123 | 0.72 | 7 |
| philopota_nigroaenea | 0 | YA | 98 | 92 | 0.984 | 6 |
| pidonia_signifera | 0 | IU | 114 | 280 | 0.763 | 3 |
| pidonia_signifera | 0 | K1 | 91 | 190 | -0.376 | 3 |
| pidonia_signifera | 0 | K2 | 91 | 133 | 0.51 | 2 |
| pieris_rapae | 1 | HE | 26 | 61 | 0.578 | 2 |
| pieris_rapae | 1 | IU | 114 | 280 | -0.103 | 2 |
| pieris_rapae | 1 | KK | 113 | 123 | -0.925 | 7 |
| pieris_rapae | 1 | PR | 89 | 90 | -0.825 | 2 |
| prothemus_ciusianus | 0 | IU | 114 | 280 | 0.29 | 4 |
| prothemus_ciusianus | 0 | K1 | 91 | 190 | -0.807 | 4 |
| prothemus_ciusianus | 0 | YA | 98 | 92 | 0.443 | 2 |
| ruizantheda_mutabilis | 1 | A1 | 69 | 49 | 0.399 | 2 |
| ruizantheda_mutabilis | 1 | VM | 10 | 11 | 0.149 | 4 |
| ruizantheda_mutabilis | 1 | VU | 11 | 12 | 0.645 | 3 |
| scaeva_melanostoma | 0 | A1 | 69 | 49 | -0.148 | 32 |
| scaeva_melanostoma | 0 | A2 | 34 | 30 | -0.668 | 15 |
| scaeva_melanostoma | 0 | A3 | 26 | 10 | 0.142 | 9 |
| sphaerophoria_menthastri | 0 | IU | 114 | 280 | 0.216 | 8 |
| sphaerophoria_menthastri | 0 | K1 | 91 | 190 | -0.098 | 9 |
| sphaerophoria_menthastri | 0 | K2 | 91 | 133 | -0.614 | 2 |
| sphaerophoria_menthastri | 0 | KK | 113 | 123 | 0.155 | 4 |
| sphaerophoria_macrogaster | 0 | IU | 114 | 280 | -0.242 | 13 |
| sphaerophoria_macrogaster | 0 | K1 | 91 | 190 | -0.733 | 2 |
| sphaerophoria_macrogaster | 0 | KK | 113 | 123 | 0.884 | 14 |
| sphaerophoria_macrogaster | 0 | YA | 98 | 92 | -0.674 | 4 |
| syritta_pipiens | 0 | K1 | 91 | 190 | 0.603 | 2 |
| syritta_pipiens | 0 | KK | 113 | 123 | 0.545 | 3 |
| syritta_pipiens | 0 | SL | 13 | 80 | -0.65 | 4 |
| syrphus_vitripennis | 0 | IU | 114 | 280 | -0.097 | 8 |
| syrphus_vitripennis | 0 | K1 | 91 | 190 | -0.009 | 4 |
| syrphus_vitripennis | 0 | K2 | 91 | 133 | -0.212 | 11 |
| syrphus_octomaculatus | 0 | SR | 26 | 59 | 0.556 | 2 |
| syrphus_octomaculatus | 0 | VM | 10 | 11 | -0.214 | 2 |
| syrphus_octomaculatus | 0 | VU | 11 | 12 | -0.15 | 3 |
| vespa_xanthoptera | 0 | IU | 114 | 280 | 0.796 | 10 |
| vespa_xanthoptera | 0 | K1 | 91 | 190 | -0.512 | 7 |
| vespa_xanthoptera | 0 | KK | 113 | 123 | -0.24 | 12 |
| vespula_germanica | 0 | CL | 94 | 115 | 0.556 | 7 |
| vespula_germanica | 0 | VM | 10 | 11 | -0.237 | 2 |
| vespula_germanica | 0 | VU | 11 | 12 | -0.25 | 3 |
